# Nutritional profiling and sensory characterization of gluten-free, high-protein, low glycemic index of sorghum-soy baked and fried chips

**DOI:** 10.3389/fnut.2025.1671158

**Published:** 2025-09-26

**Authors:** Zara Fatima, Beenish Israr, Nizwa Itrat, Abdul Momin Rizwan Ahmad

**Affiliations:** ^1^Food Compositional Analytical Laboratory, Institute of Home Sciences, University of Agriculture, Faisalabad, Pakistan; ^2^Department of Health Sciences, University of York, York, United Kingdom; ^3^Department of Human Nutrition and Dietetics, NUST School of Health Sciences, National University of Sciences & Technology (NUST), Sector H-12, Islamabad, Pakistan

**Keywords:** nutritional profiling, gluten-free, high-protein, sorghum, soy, sustainable diets, sustainable food systems

## Abstract

**Introduction:**

Wheat production faces increasing threats from climate change, highlighting the need for resilient, nutrient-dense, and sustainable alternatives such as sorghum and soybeans.

**Objective:**

The objective of the current study was to develop high-protein, low-glycemic, sustainable sorghum-soy snacks. The novelty of this study lies in providing a healthy snack that is gluten-free, rich in protein, and has a low glycemic index.

**Methods:**

In this study, sorghum-soy chips were prepared using four formulations with varying proportions of soybean and sorghum flours (T0: 50:50, T1: 55:45, T2: 60:40, and T3: 65:35) and processed through baking and frying techniques. The nutritional composition, mineral profile, sensory characteristics, and glycemic index of the composite flour were evaluated to determine their functional and health-promoting potential. All the values obtained from different analyses of chips were subjected to a one-way analysis of variance (ANOVA) analysis through the software Statistix 8.1.

**Results:**

The sorghum-soy chips developed in this study demonstrated significant nutritional enhancement with increasing soybean incorporation. Proximate analysis revealed a marked (*p* < 0.05) increase in protein content, particularly in T3 (65:35 soybeans: sorghum ratio), while mineral analysis indicated appreciable levels of calcium (196.70 mg/100 g), iron (13.61 mg/100 g in T3), and magnesium (250.96 mg/100 g) among fried treatments, highlighting the potential to address micronutrient deficiencies. Sensory evaluation showed that T_1_ (55:45) achieved the highest scores for color, flavor, crispiness, aroma, and overall acceptability, suggesting strong consumer preference. Furthermore, baked chips displayed a lower glycemic index and reduced fat content compared to fried variants, making them more appropriate for health-conscious and diabetic individuals. In contrast, fried chips exhibited superior texture and shelf stability, emphasizing their appeal in terms of sensory quality.

**Conclusion:**

Sorghum-soy chips demonstrated potential as gluten-free, protein-rich, low-glycemic index snacks that promote dietary diversity and support efforts to combat malnutrition. This study provides insights into creating healthier snack options using climate-resilient crops while adhering to sustainable food system principles.

## Graphical abstract

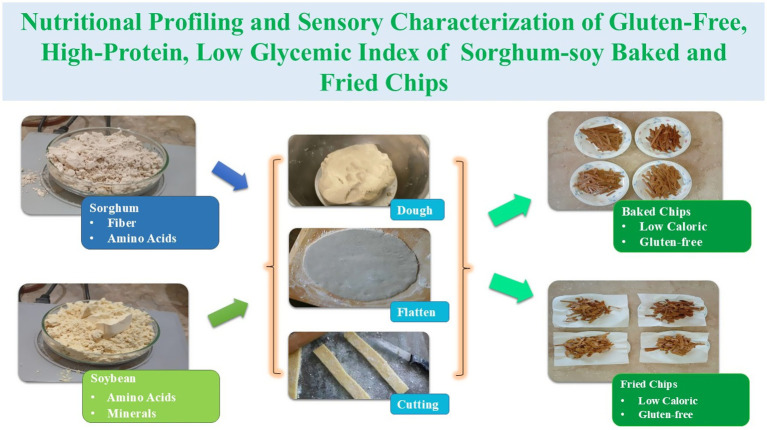

## 1 Introduction

Wheat has historically been a major source of calories for humans, providing nearly 20% of global dietary energy needs (1). However, rising population pressures and the limitations of agroecosystems have made it difficult for wheat production to keep pace with global demand. Climate change, soil depletion, droughts, and extreme heat events have further threatened monoculture wheat systems (2). In the United States, extended weather phenomena like flash droughts have resulted in up to a 25% reduction in winter wheat yields, with similar declines observed in other major producing regions (3). These vulnerabilities highlight the risks of over-reliance on a single staple crop and the growing gap between wheat supply and global demand. Addressing these challenges requires a shift toward diverse, nutrient-dense, and climate-resilient crops (4).

Alternative grains such as sorghum, millet, quinoa, and amaranth, along with legumes such as soybeans, offer sustainable options for strengthening food systems. These crops are naturally gluten-free, highly adaptable to marginal soils, and rich in slow-digesting carbohydrates, dietary fiber, phytochemicals, and quality proteins (5). Their inclusion in modern diets could enhance ecological resilience, reduce chronic disease risks, and provide sustainable food alternatives amidst global uncertainties (6).

Prior studies have shown that alternative grains and legumes can significantly enhance the nutritional and functional properties of gluten-free foods (7, 8). For example, Iftikhar et al. (7) reported that iron- and zinc-fortified chickpea snacks improved protein intake and cognitive performance in preadolescents. In a recent study, gluten-free snack bars were formulated by incorporating dates and pseudo cereals, which boosted protein, fiber, and antioxidant content, and were well accepted by gluten-intolerant consumers (9). Similarly, the increasing demand for gluten-free alternatives in Pakistan is due to changing dietary habits and rising gluten sensitivities. In these findings, this study proposes a sorghum-soy snack that improves protein, fiber, and micronutrient content, thereby promoting health and sustainability (10).

Soybeans (*Glycine max*) are a nutrient-dense legume, containing 37.7% protein with a complete amino acid profile and 28.2% healthy lipids, including omega-3 and omega-6 fatty acids (11). Soy provides 469.8 kcal/100 g and 5.4% dietary fiber and is an excellent source of minerals such as calcium (300.4 mg), magnesium (258.2 mg), and iron (16.4 mg). Isoflavones in soy act as phytoestrogens with antioxidant activity, supporting bone health, hormonal balance, and cardiovascular function (12). Agriculturally, soy enhances soil fertility through nitrogen fixation, reducing dependence on synthetic fertilizers and associated greenhouse gas emissions (13).

Sorghum (*Sorghum bicolor*), a gluten-free cereal, is highly resilient to drought and heat stress, making it ideal for cultivation in arid regions (14). It contains approximately 11% protein, 3.5% fat, and 68–72% carbohydrates, along with 6–8 g/100 g of dietary fiber. Sorghum is also rich in polyphenols and tannins, which contribute antioxidant and glycemic-lowering properties (15). Its slow-digesting starch and low glycemic index make it suitable for diabetes management and weight control (16). Together, sorghum and soy complement each other’s nutritional profiles, providing balanced macronutrients and amino acids for improved health outcomes and environmental sustainability (17).

Celiac disease (CD) affects 0.75–1.6% of the global population, with biopsy-confirmed prevalence ranging from 0.3–1.0% (15). Non-celiac gluten sensitivity (NCGS) impacts an estimated 6–10% of individuals globally, with regional variations. Recent data from Pakistan indicate that CD affects 0.6–1.0% and NCGS 2–5% of individuals, resulting in a combined burden of 2.6–6.0% of the population (18). These statistics underscore the growing need for nutrient-rich, gluten-free products that address dietary restrictions and enhance public health (19).

Climate change continues to pose a considerable and escalating danger to global wheat production, making the promotion of alternative crops like sorghum and soy an essential strategy in ensuring food security. The connection is direct: as climate-related risks to wheat are increasing in intensity, sorghum and soy appear as more viable, and thus more sustainable, alternatives, due to inherent their climate-resilient characteristics.

### 1.1 Climate change and wheat production challenges

Wheat is especially vulnerable to climate change among the major commodities that sustain global food security. It is one of the crops that is very sensitive to the minutest of environmental conditions under which it grows and produces. Wheat is most susceptible to high temperatures during critical development stages, especially grain filling. These extreme heats shorten grain filling, causing smaller, less dense grains and, hence, reducing yields. Wheat production greatly depends on the provision of adequate rainfall or irrigation. With decreasing water from climate change, the drought effects directly jeopardize rain-fed wheat crops and raise the costs of irrigated production. Increasing frequency of extreme weather, such as floods or unseasonal frosts, could trigger a disastrous situation, leading to total crop failure in particular localized regions (20).

### 1.2 Sorghum as an alternative crop with climate resilience

The deep fibrous root system creates an exceptional barrier against water loss by sorghum, coupled with the thick waxy covering of the leaves. Sorghum, being a C4 plant for photosynthesis, employs sunlight for energy conversion with utmost efficiency even under hot and dry conditions. This gives it an obvious yield advantage over wheat in arid and semi-arid conditions. Lower water and fertilizer requirements, combined with lesser environmental and economic cost to the producer from a sorghum-planting point of view than wheat. Apart from being a food source for people who cannot tolerate gluten, sorghum is an important animal feed and biofuels crop, giving multiple market opportunities that can stabilize farmer income (21).

Soybean is a valuable alternative, complementing sorghum benefits, particularly in a rotation or mixed cropping system. Soybeans, being legumes, fix atmospheric nitrogen into the soil through a symbiotic relationship with bacteria. This enriches the soil so that nitrogen fertilizers need not be applied, benefiting subsequent crops, including sorghum in the rotation. Together, they not only improve soil health over the long term but also diminish the carbon footprint of fertilizer production. Adaptable to a wide variety of climates, soy is a major source of protein and oil. The dietary importance, especially the high protein content of soy, helps in meeting world nutritional needs at this time when heat-stressed wheat may decline in protein quality. The introduction of soy and sorghum into the traditional wheat-based agricultural system creates varied income sources, thus protecting the farmer from market volatility and production risks of a single crop. Sorghum and soy promotion would place agricultural policy and research ahead of the game in combating climate change challenges to global food security. These crops would not stand in for wheat; they are seen as a strategic shift toward a truly resilient, sustainable, and diversified agricultural system (22).

This study aims to develop and evaluate high-protein, low-glycemic sorghum-soy snacks to support dietary diversity and health promotion. The objectives include determining the nutritional composition of gluten-free snacks, optimizing sorghum and soybean flour blends, and assessing the glycemic index, storage stability, and sensory acceptability of the developed products. By leveraging the nutritional synergy of sorghum and soy, this research seeks to create functional snacks that meet global health and sustainability targets. Sorghum contains sulfur-containing amino acids like methionine and cysteine, which complement the amino acid profile of soybean, making it an excellent source of complete protein. Sorghum contains high levels of slowly digestible starch and resistant starch, which gives it a low glycemic index, and when combined with high protein-containing soy, it makes it a perfect product (23). This sorghum-soy combination offers a unique advantage in producing a low glycemic index product, a property that is less pronounced in wheat-chickpea or corn-bean combinations (24).

Nutrient synergy and bioactivity are the synergistic effects of the antioxidant-rich sorghum (phenolic compounds and tannins) combined with beneficial isoflavones and healthful fats from soybeans. This creates a potent mix of functional properties beyond protein enrichment, and in such a product, one can expect potential antidiabetic and cardiovascular health benefits.

It is worth noting that the sorghum-soy blend is naturally gluten-free and may thus be safely consumed in cases of celiac disease or gluten sensitivity, a significant advantage in the current market, which contains no wheat or any other gluten-containing grains (25).

## 2 Materials and methods

### 2.1 Materials

Based on the experimental design, the following materials were used in this study. Composite flours were prepared using soybean (*Glycine max*) and sorghum (*Sorghum bicolor*) grains, both sourced from the local grain market in Faisalabad, Pakistan. Sorghum grains were carefully sorted to remove extraneous impurities, thoroughly washed, and sun-dried to reduce surface moisture. To ensure uniform drying and minimize residual moisture content, the grains were oven-dried at 80 °C for 2–3 min. Soybean seeds underwent the same cleaning, washing, sun-drying, and oven-drying procedures. Both sorghum and soybean grains were ground using a hammer mill and sieved through a fine mesh screen to achieve a uniform particle size. The resulting flours were passed through muslin cloth to remove coarse particles before blending them in predetermined ratios for sorghum-soy chip formulations. Commercially available cooking oil (purchased locally in Faisalabad) was utilized for frying treatments. Other dry ingredients, including gram flour, salt, and baking soda, were procured from SB Store in Faisalabad to prepare the dough for chips. Analytical-grade chemicals and reagents required for proximate composition, phytochemical evaluation, antioxidant activity, and mineral analysis were obtained from certified scientific suppliers in Faisalabad, Pakistan. These included solvents and compounds such as methanol, ethanol, hydrochloric acid, sulfuric acid, sodium hydroxide, petroleum ether (boiling point: 30–60°C), n-hexane, ammonium sulfate, anhydrous sodium sulfate, potassium iodide, and trichloromethane. All reagents were of analytical grade and used without further purification.

### 2.2 Preparation of composite flours

The treated sorghum and soybean flours were mixed in specific proportions to produce the composite flour blends needed to make sorghum-soy chips, which had the desired nutritional and functional qualities. Before the dough was prepared and the product was developed, the blending was done by hand to guarantee homogeneity. Glycemic index was calculated by using the weighted average formula for composite flours of sorghum and soy composite (26).

### 2.3 Formulation of sorghum-soy chips

To make the dough, composite flours were combined in a mixing utensil with gram flour, baking soda, oil, and salt. After kneading the dough until it was uniform, it was flattened out into thin sheets and cut into uniformly sized and shaped chips according to the treatment plan (Table 1). Baking and frying were the two cooking techniques used. Chips for the baked version were baked for 5–7 min at 150°C, chilled, and then placed in airtight containers (27). For the fried version, oil was heated to 130°C, and the chips were fried for 2–3 min, allowing for a controlled temperature drop of 10°C. Fried chips were placed on tissue paper to absorb excess oil for 60 s before storing (28).

**Table 1:** Treatment plan of sorghum-soy baked and fried chips.

A. Baked sorghum-soy chips			B. Fried sorghum-soy chips			Glycemic index of composite flour
Treatments	Soybean (%)	Sorghum (%)	Treatments	Soybean (%)	Sorghum (%)	
T_0_	50	50	T_0_	50	50	42.51
T_1_	55	45	T_1_	55	45	39.75
T_2_	60	40	T_2_	60	40	37.02
T_3_	65	35	T_3_	65	35	34.25

### 2.4 Analysis of raw materials of sorghum-soy chip

The proximate (moisture, ash, fat, fiber, protein, and nitrogen-free extract (NFE)) composition of food samples was determined using standard McCleary et al. (84) methods. Moisture content was analyzed by drying samples in a hot air oven at 105°C for 24 h until a constant weight was achieved, followed by cooling in a desiccator and weighing (29). Ash content was determined by incinerating weighed samples in a muffle furnace at 550°C until a light grayish residue was formed, and the ash percentage was calculated (30). The Soxhlet extraction method was employed to determine crude fat, using petroleum ether as a solvent, with extraction carried out for 4–5 h. After solvent removal, residues were oven-dried at 100°C and weighed (31). Crude fiber content was assessed by sequentially treating samples with 1.25% sulfuric acid and 1.25% sodium hydroxide, followed by filtration, washing, drying, and ashing. The residue weight differences provided the fiber content (32). Crude protein was measured using the Kjeldahl method, involving digestion with H₂SO₄ and catalysts, distillation, and titration with H₂SO₄. Nitrogen content was then converted to protein by multiplying by 6.25 (33). Nitrogen-free extract (NFE) was calculated by subtracting the sum of moisture, ash, protein, fat, and fiber from 100% (34).

#### 2.4.1 Total phenolic and flavonoid content

The method used by Consumi et al. (35) was utilized to measure the total flavonoid content (TFC), which involved combining the methanolic extracts of the sample with distilled water, 10% AlCl₃, and 5% NaNO₂ and then incubating the mixture. To determine the amounts of flavonoids, absorbance was measured at 510 nm using a spectrophotometer (83). By reacting extracts with Na₂CO₃ and the Folin–Ciocalteu reagent, the Folin–Ciocalteu method was used to calculate the total phenolic content (TPC). Following incubation, findings were reported in terms of phenolic content using absorbance measured at 725 nm.

#### 2.4.2 Antioxidant analysis

The 2,2-diphenyl-1-picrylhydrazyl (DPPH) technique of scavenging free radicals was used to determine the overall antioxidant activity. To make the extracts, 0.5 g of the material was combined with 5 mL of 80% methanol and shaken for 2 h. Then, 25 mL of the extract was mixed with a 2-mg DPPH solution in 50 mL of methanol, and the mixture was left in the dark to finish the reaction. At 515 nm, absorbance was measured with a spectrophotometer (36).

#### 2.4.3 Mineral analysis

Using an atomic absorption spectrophotometer and the Association of Official Analytical Communities International (85)method, the mineral content (Ca, Fe, and Mg) was ascertained. Concentrated HNO₃ and HClO₄ were used to digest samples (0.5 g) at controlled temperatures (85–180°C) until yellow fumes emerged. After filtering and diluting the digested solution with 100 mL of distilled water, the mineral concentrations were measured and converted from ppm to mg (37).

### 2.5 Analysis of sorghum-soy chips

The methods of AOAC (2019) were used to determine the proximate composition of sorghum-soy chips, which included moisture, ash, crude fat, fiber, protein, and nitrogen-free extract (38). Total flavonoid and phenolic contents were analyzed according to the method used by Domínguez-Hernández et al. (39). Antioxidant activity of the chips was evaluated using the DPPH assay, as described by Vasisht et al. (40). According to AOAC recommendations, the mineral content (Ca, Fe, and Mg) was measured using an atomic absorption spectrophotometer (AOAC, 2019). The created chips’ thorough nutritional and functional profile was guaranteed by these assessments.

### 2.6 Sensory evaluation

For the sensory evaluation of sorghum-soy chips, 10 trained panelists (aged 20–40 years) from the Institute of Home Sciences, University of Agriculture, Faisalabad, Pakistan, participated. Panelists were selected based on their background in food science and trained to recognize and score sensory attributes. Before testing, they were briefed on study objectives, evaluation procedures, and sensory parameters. The chips were assessed for color, flavor, crispiness, aroma, and overall acceptability using a 9-point hedonic scale (1 = strongly dislike, 9 = like extremely) (41). Each sample was randomly coded and served in odorless transparent bowls under standardized conditions. Panelists rinsed their mouths with tasteless, odorless water between samples to prevent sensory bias and provided qualitative feedback to support quantitative scoring.

### 2.7 Treatment plan

The study utilized four treatment formulations of sorghum-soy chips prepared by varying soybean and sorghum flour ratios (T_0_: 50:50, T_1_: 55:45, T_2_: 60:40, and T_3_: 65:35). These formulations were processed using two cooking methods, baking and frying, to assess their impact on nutritional, sensory, and functional properties (Table 1). The design allowed for a comparative evaluation of how soybean enrichment and cooking techniques influenced the quality of sorghum-soy chips.

### 2.8 Statistical analysis

All experimental data were analyzed using a one-way analysis of variance (ANOVA) with a general linear model approach, Statistix 8.1 (Analytical Software, Tallahassee, FL, United States). When ANOVA indicated significant differences (*p* < 0.05), Tukey’s *post hoc* test was applied to determine pairwise treatment differences (42). The results were expressed as mean ± standard deviation (SD). To enhance the reliability of findings, all measurements were performed in triplicate. A significance level of *p* of < 0.05 was considered statistically significant throughout the analysis.

## 3 Results and discussion

### 3.1 Chemical composition and nutritional value of raw materials

The proximate composition of raw sorghum and soybean flours, showing significant differences (*p* < 0.05) in moisture, ash, crude fat, crude fiber, crude protein, and nitrogen-free extract (NFE), is presented in Table 2. Soybean flour exhibited slightly higher moisture (8.65%) than sorghum flour (7.99%), which could increase susceptibility to spoilage and emphasizes the importance of proper storage. Similarly, these values are marginally higher than those published by Iwayemi and Ikujenlola (43). Ash content was significantly higher in soybean (4.01%) than in sorghum (1.68%), reflecting its superior mineral density. These findings align with those in the study by Adekiya et al. (44), who reported comparable ash values for soybeans (3.98%), and with those in the study by Jenfa et al. (45) for sorghum (1.57%). Crude fat was substantially higher in soybean (27.35%) than in sorghum (3.00%) due to its oilseed nature, consistent with the findings of the study by Onaolapo et al. (46), highlighting the predominance of polyunsaturated fatty acids in soy. Soybeans also contained higher crude fiber (5.77%) than sorghum (1.80%), supporting their functional food potential for improving gut health and glycemic control. The crude fiber values reported by Joseph et al. (47) for various soybean cultivars ranged from 5.60 to 5.85%, which is in good accord with these data. In support of the current findings, Mohapatra et al. (48) observed a similar fiber value of 1.75% in fermented sorghum. Protein content was significantly higher in soybean (38.07%) than in sorghum (10.56%), confirming its rich amino acid profile, as observed by Okwunodulu et al. (49) and Pontieri et al. (50). In contrast, sorghum displayed higher NFE (74.31%) compared to soybean (16.81%), positioning it as an important energy source in cereal-based formulations. Imafidon (51) reported 16.65% NFE in soybean flour, while Adebayo and Oladunjoye (52) found that sorghum NFE ranged between 72.80–75.10%, confirming these results. These findings suggest that blending sorghum and soybean flour can improve nutritional profiles for functional food development. The glycemic index of T3 composite flour is 34.25, while the highest was T0, which was 42.51 (Table 1).

**Table 2:** Results of proximate composition (%) of soybean and sorghum flour.

Parameters	Soybean (mean ± SD)	Sorghum (mean ± SD)
Moisture	7.9 ± 0.28	8.65 ± 0.38
Ash	4.01 ± 0.18	1.68 ± 0.074
Crude fat	27.35 ± 1.11	3.00 ± 0.11
Crude fiber	5.77 ± 0.22	1.80 ± 0.08
Crude protein	38.07 ± 1.7	10.56 ± 0.48
NFE	16.81 ± 0.7	74.31 ± 3.60
Glycemic index	15	70

### 3.2 Total phenolic and flavonoid content

The sorghum flour exhibited significantly higher total phenolic content (TPC: 623 milligrams of gallic acid equivalents per gram (mg GAE/100 g)) and total flavonoid content (TFC: 101 mg QE/100 g) compared to soybean flour (TPC: 2.61 mg GAE/100 g; TFC: 0.81 mg QE/100 g), as shown in Table 3. Singh et al. (53) reported TPC and TFC values of 131 mg GAE/100 g and 5,700 mg QE/100 g, respectively, in native sorghum varieties, highlighting varietal and processing influences. Conversely, Đurović et al. (54) documented lower antioxidant activity in soybean flour due to its biochemical focus on lipid and protein synthesis. These findings emphasize sorghum’s antioxidant potential in functional food formulations.

**Table 3:** Results of phytochemical screening test of soybean and sorghum flour.

Parameters	Soybean (mean ± SD)	Sorghum (mean ± SD)
TPC (mg GAE/100 g)	2.61 ± 0.10	623 ± 25.11
TFC (mg QE/100 g)	0.81 ± 0.03	101 ± 4.99

### 3.3 Antioxidant analysis

Sorghum flour demonstrated significantly higher DPPH radical scavenging activity (3,900 μmol TE/100 g) compared to soybean flour (351 μmol TE/100 g) (*p* < 0.05), as shown in Table 4. This enhanced antioxidant capacity is attributed to sorghum’s rich phenolic content, including flavonoids and tannins. Mumeen et al. (55) observed similar trends, reporting low DPPH activity in soybean lecithin due to its limited polyphenolic profile. Collins et al. (56) reported DPPH values of 4,200–4,650 μmol TE/100 g in raw sorghum, emphasizing varietal and processing effects. These findings highlight sorghum’s potential for enhancing oxidative stability in functional food systems.

**Table 4:** Results of DPPH (μmol TE/100 g) of soybean and sorghum flour.

Parameters	Soybean	Sorghum
DPPH	351 ± 0.12	3,900 ± 0.02

### 3.4 Mineral analysis

Soybean flour exhibited significantly higher calcium (305.16 mg/100 g), iron (15.7 mg/100 g), and magnesium (280 mg/100 g) levels compared to sorghum (27.98, 4.4, and 165 mg/100 g, respectively, *p* < 0.05). Comparable mineral levels in soy were reported by Javed et al. (57), while Mystkowska et al. (58) documented lower values for sorghum (Table 5). These findings emphasize soybean’s superior mineral density and sorghum’s contribution to magnesium, supporting their integration into functional food formulations.

**Table 5:** Results of mineral analysis (mg/100 g) of soybean and sorghum flour.

Parameters (mg/100 g)	Soybean (mean ± SD)	Sorghum (mean ± SD)
Ca	305.16 ± 10.05	27.98 ± 1.17
Fe	15.7 ± 1.5	4.4 ± 1.04
Mg	280 ± 20.64	165 ± 10.25

### 3.5 Chemical composition and nutritional value of baked sorghum-soy chips

The proximate composition of baked sorghum-soy chips, showing significant variations (*p* < 0.05) in moisture, ash, crude fat, crude fiber, crude protein, and nitrogen-free extract (NFE) across treatments, presents in Table 6. Moisture content ranged from 2.45% (T0) to 3.16% (T3), with the highest level in T3 (65% soy + 35% sorghum). This increase may be attributed to soy proteins’ hydrophilic amino acid residues, which enhance water-binding and retention during baking. Mananda et al. (59) reported higher moisture values (3.66–3.73%) in sorghum-based high-protein crackers, possibly due to differences in formulation and product structure. The lower moisture in chips supports crispness and extends shelf life. Ash content significantly increased from 3.36% (T0) to 3.90% (T3), reflecting the soybean’s mineral-rich composition. In contrast, Raza et al. (60) found lower ash values (1.25–1.62%) in soybean-wheat cookies, likely due to the refining of wheat flour. Fat content increased significantly (*p* < 0.05), ranging from 11.55% in T0 to 14.10% in T3, consistent with soybeans’ lipid-rich profile demonstrated by Mouafo et al. (61). Crude fiber slightly declined from 6.98% (T0) to 6.64% (T3), reflecting soybean’s lower fiber levels compared to sorghum (62). Protein content increased significantly (*p* < 0.05) from 24.77 to 25.59%, confirming soy’s contribution to nutritional quality demonstrated by Tsegba et al. (63). NFE content showed a slight but significant increase (42.05–42.45%), likely due to residual carbohydrates in the flours. Pallavi et al. (64) found that his crackers had relatively higher quantities of total carbohydrates, ranging from 54.17 to 68.03%, which may be due to the Maillard reactions (65). These findings support the development of nutrient-dense, functional baked chips to address malnutrition and promote dietary diversity.

**Table 6:** Results of proximate analysis (%) of baked sorghum-soy chips of different treatments.

Parameters	T_0_	T_1_	T_2_	T_3_
Moisture	2.45 ± 0.04^d^	2.73 ± 0.04^c^	2.94 ± 0.05^b^	3.16 ± 0.08^a^
Ash	3.36 ± 0.05^c^	3.57 ± 0.10^bc^	3.70 ± 0.06^ab^	3.90 ± 0.10^a^
Fat	11.55 ± 0.11^d^	12.44 ± 0.13^c^	13.25 ± 0.11^b^	14.10 ± 0.11^a^
Fiber	6.98 ± 0.03^a^	6.83 ± 0.02^b^	6.74 ± 0.02^c^	6.64 ± 0.02^d^
Protein	24.77 ± 0.09^c^	24.95 ± 0.06^bc^	25.08 ± 0.07^b^	25.59 ± 0.13^a^
NFE	42.05 ± 0.04^c^	42.22 ± 0.04^b^	42.31 ± 0.05^b^	42.45 ± 0.05^a^

The mean values carrying the same letters in a column are not significantly different.

#### 3.5.1 Total phenolic and flavonoid content

Significant differences (*p* < 0.05) were observed in total phenolic content (TPC: 273.42–344.62 mg GAE/100 g) and total flavonoid content (TFC: 28.98–37.58 mg QE/100 g) across treatments (Table 7). T3 exhibited the highest TPC due to sorghum’s heat-stable phenolic compounds, such as 3-deoxyanthocyanidins and phenolic acids. These values are comparable to acha–chia–soy cake blends demonstrated by Ogunnowo et al. (66), though TFC was relatively lower, possibly due to compositional differences. The synergistic presence of soybean isoflavones and sorghum polyphenols enhanced antioxidant capacity, underscoring the influence of formulation and thermal processing on phytochemical retention.

**Table 7:** Results of phytochemical screening of baked sorghum-soy chips of different treatments.

Parameter	T_0_	T_1_	T_2_	T_3_
TPC (mg GAE/100 g)	338.66 ± 1.52^a^	311.66 ± 3.51^b^	284.66 ± 5.50^c^	262.66 ± 2.51^d^
TFC (mg QE/100 g)	63.35 ± 01.49^a^	55.35 ± 04.47^ab^	51.11 ± 50.15^bc^	46.01 ± 45.02^c^

The mean values carrying the same letters in a column are not significantly different.

#### 3.5.2 Antioxidant analysis

Baked sorghum-soy chips demonstrated higher DPPH activity (241.95–315.89 μmol TE/100 g) (Table 8). Compared to sorghum-banana snack bars (5.00–15.05%) reported by Setyaningtiyas et al. (67). This enhanced antioxidant capacity could be attributed to heat-stable phenolics in sorghum and soy isoflavones. Baking likely promoted the release of bound polyphenols, improving radical scavenging activity. In contrast, the lower antioxidant potential of banana flour highlights the functional advantage of sorghum-soy formulations in developing health-promoting snacks.

**Table 8:** Results of DPPH (μmol TE/100 g) value in baked sorghum-soy chips of different treatments.

Parameter	T_0_	T_1_	T_2_	T_3_
DPPH	241.95 ± 243.03^d^	266.88 ± 268.37^c^	294.12 ± 296.33^b^	315.89 ± 317.56^a^

The mean values carrying the same letters in a column are not significantly different.

#### 3.5.3 Mineral analysis

As shown by Table 9, baked sorghum-soy chips showed significantly higher calcium (156.94–196.08 mg/100 g), iron (10.11–11.89 mg/100 g), and magnesium (224.97–249.05 mg/100 g) than fortified sorghum (2.99–3.99, 1.98–2.53, and 0.39–0.57 mg/100 g) (68). This difference can be attributed to soy’s mineral richness, reduced moisture from baking, and minimal nutrient losses, establishing baked sorghum-soy chips as nutrient-dense functional alternatives to conventional fortified cereal products.

**Table 9:** Results of mineral analysis (mg/100 g) of baked sorghum-soy chips of different treatments.

Parameter (mg/100 g)	T_0_	T_1_	T_2_	T_3_
Ca	156.94 ± 158.42^c^	171.90 ± 174.20^b^	183.16 ± 185.08^ab^	196.08 ± 198.14^a^
Fe	10.11 ± 10.13^d^	10.82 ± 10.89^c^	11.23 ± 11.25^b^	11.89 ± 11.93^a^
Mg	224.97 ± 225.79^c^	231.94 ± 233.17^bc^	241.42 ± 243.89^ab^	249.05 ± 249.71^a^

The mean values carrying the same letters in a column are not significantly different.

### 3.6 Chemical composition and nutritional value of fried sorghum-soy chips

The proximate composition of fried sorghum-soy chips, with significant differences (*p* < 0.05) observed across treatments. Moisture content ranged from 2.90% (T_0_: 50% soy, 50% sorghum) to 3.60% (T_3_: 65% soy, 35% sorghum), as shown in Table 10. These values were considerably lower than the 10.50–10.91% reported by Izuakor et al. (69) for gluten-free blends like Cerelac, likely due to rapid surface water evaporation during frying, which enhances crispness and shelf life. Ash content increased from 3.31% in T0 to 3.85% in T3, reflecting the mineral-rich composition of soybean and sorghum flours. This finding aligns with that in the study by El-Hadidy et al. (70), though their gluten-free crackers showed lower ash levels (0.713–2.039%) due to formulation differences. Fat content increased significantly (22.08–26.72%), which was consistent with the findings of Sharma et al. (71), who observed increased lipid retention post-frying (25.07–28.97%). Controlled oil absorption in this study minimized excessive uptake compared to other formulations. Crude fiber decreased slightly (5.63–5.05%) as soybean replaced sorghum, which naturally has higher fiber content. Dehideniya et al. (72) revealed an even lower fiber content (2.18%) in their cracker recipe, most likely because of the lack of whole-grain fractions and the preponderance of low-fiber additives. Protein content increased markedly from 24.32% in T0 to 32.87% in T3 (*p* < 0.05), surpassing levels in plantain–sorghum crackers fortified with okara (73). Conversely, nitrogen-free extract (NFE) declined (41.60–32.47%) as protein-rich soybean displaced carbohydrate-dense sorghum, mirroring the macronutrient shift. In contrast, El Feky et al. (74) noted that crackers enhanced with leafy vegetable powders had greater carbohydrate values (65.12–72.57%), which are probably due to their wheat flour base, which naturally raises the starch level. These results demonstrate the potential of fried sorghum-soy chips as nutrient-dense functional snacks for protein–energy malnutrition interventions.

**Table 10:** Results of proximate analysis (%) of fried sorghum-soy chips of different treatments.

Parameters	T_0_	T_1_	T_2_	T_3_
Moisture	2.90 ± 2.94^b^	3.25 ± 3.33^ab^	3.45 ± 3.53^a^	3.60 ± 3.67^a^
Ash	3.31 ± 3.33^c^	3.50 ± 3.52^bc^	3.65 ± 3.67^b^	3.85 ± 3.88^a^
Fat	22.08 ± 22.24^c^	23.83 ± 24.13^bc^	25.68 ± 26.14^ab^	26.72 ± 27.08^a^
Fiber	5.63 ± 5.62^a^	5.44 ± 5.43^b^	5.24 ± 5.21^c^	5.05 ± 5.01^d^
Protein	24.32 ± 24.46^b^	27.53 ± 28.15^ab^	30.70 ± 31.79^ab^	32.87 ± 34.10^a^
NFE	41.60 ± 41.20^a^	37.54 ± 36.89^b^	35.09 ± 34.73^bc^	32.47 ± 32.34^c^

The mean values carrying the same letters in a column are not significantly different.

#### 3.6.1 Total phenolic and flavonoid content

Phytochemical analysis (Table 11) revealed a progressive increase in total phenolic content (TPC: 286.30–344.62 mg GAE/100 g) and total flavonoid content (TFC: 28.98–37.58 mg QE/100 g) with higher soybean incorporation. These levels surpassed those reported in fiber-enriched crackers (TPC: 6.95–10.54%, TFC: 0.87–8.75 mg/100 g) by Ujong et al. (75), likely due to sorghum’s polyphenols and soybean isoflavones. Frying enhanced the extractability of bound phenolics, contributing to the superior antioxidant potential of fried sorghum-soy chips. This finding underscores the combined impact of formulation and thermal processing on bioactive compound retention.

**Table 11:** Results of phytochemical screening test of fried sorghum-soy chips of different treatments.

Parameter	T_0_	T_1_	T_2_	T_3_
TPC (mg GAE/100 g)	286.30 ± 286.73^c^	312.13 ± 315.67^b^	327.42 ± 330.23^ab^	344.62 ± 347.49^a^
TFC (mg QE/100 g)	28.98 ± 29.24^b^	33.21 ± 33.95^ab^	35.56 ± 36.21^a^	37.58 ± 38.04^a^

The mean values carrying the same letters in a column are not significantly different.

#### 3.6.2 Antioxidant analysis

The DPPH activity of fried sorghum-soy chips, shown by Table 12, increased significantly with increasing soybean levels, from 214.55 μmol TE/100 g (T0) to 283.56 μmol TE/100 g (T3) (*p* < 0.05). This improvement is attributed to the release of heat-released phenolic acids from sorghum and soy isoflavones. Comparatively, Singh et al. (76) observed lower DPPH values (24.81–55.12%) in extruded rice–lentil–chickpea–tomato snacks, which may be due to polyphenolic losses and degradation during extrusion and frying processes.

**Table 12:** Results of DPPH (μmol TE/100 g) in fried sorghum-soy chips of different treatments.

Parameter	T_0_	T_1_	T_2_	T_3_
DPPH	214.55 ± 215.90^d^	238.75 ± 240.54^c^	260.66 ± 262.64^b^	283.56 ± 286.01^a^

The mean values carrying the same letters in a column are not significantly different.

#### 3.6.3 Mineral analysis

As depicted in Table 13, fried sorghum-soy chips demonstrated significantly higher calcium (154.03–196.70 mg/100 g), iron (10.22–13.61 mg/100 g), and magnesium (223.46–250.96 mg/100 g) levels with increasing soybean addition (*p* < 0.05). These values surpass those of sprouted flour crackers reported by Negm et al. (77). The increase could be attributed to soybean’s mineral density and moisture reduction during frying, which concentrates nutrients, enhances bioavailability, and positions the chips as functional snacks for addressing mineral deficiencies.

**Table 13:** Results of mineral analysis (mg/100 g) of fried sorghum-soy chips of different treatments.

Parameter (mg/100 g)	T_0_	T_1_	T_2_	T_3_
Ca	154.03 ± 154.54^d^	168.80 ± 169.60^c^	182.94 ± 183.86^b^	196.70 ± 197.60^a^
Fe	10.22 ± 10.27^c^	11.21 ± 11.29^bc^	12.07 ± 12.13^b^	13.61 ± 13.89^a^
Mg	223.46 ± 223.78^d^	233.40 ± 234.20^c^	242.30 ± 243.23^b^	250.96 ± 251.95^a^

The mean values carrying the same letters in a column are not significantly different.

### 3.7 Sensory evaluation of sorghum-soy chips

**Figure 1:**
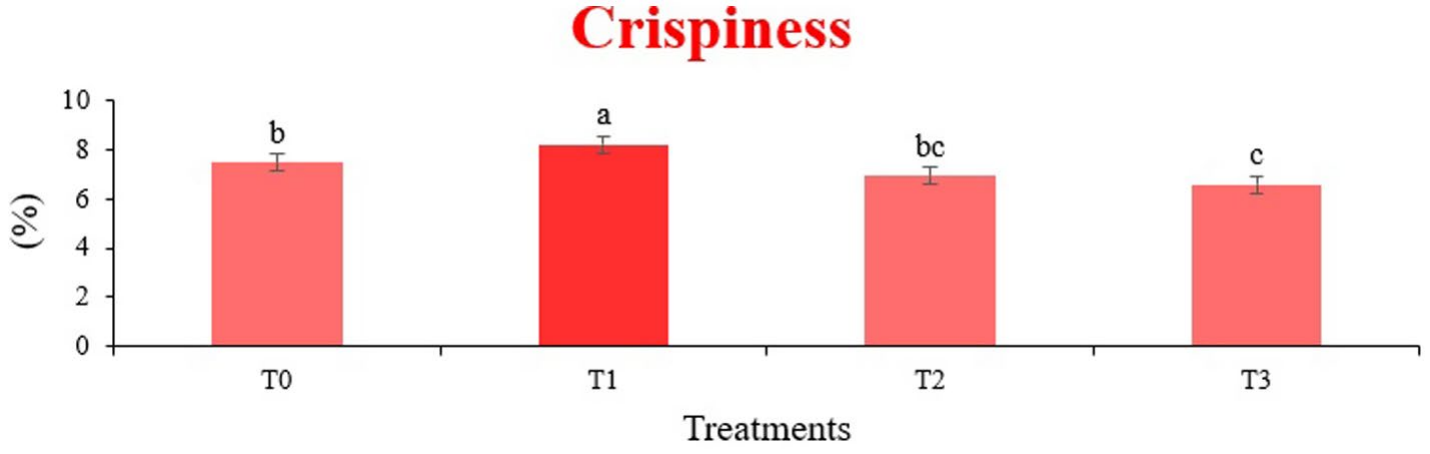
Mean results of crispiness in sensory evaluation of four different treatments of baked sorghum-soy chips.

**Figure 2:**
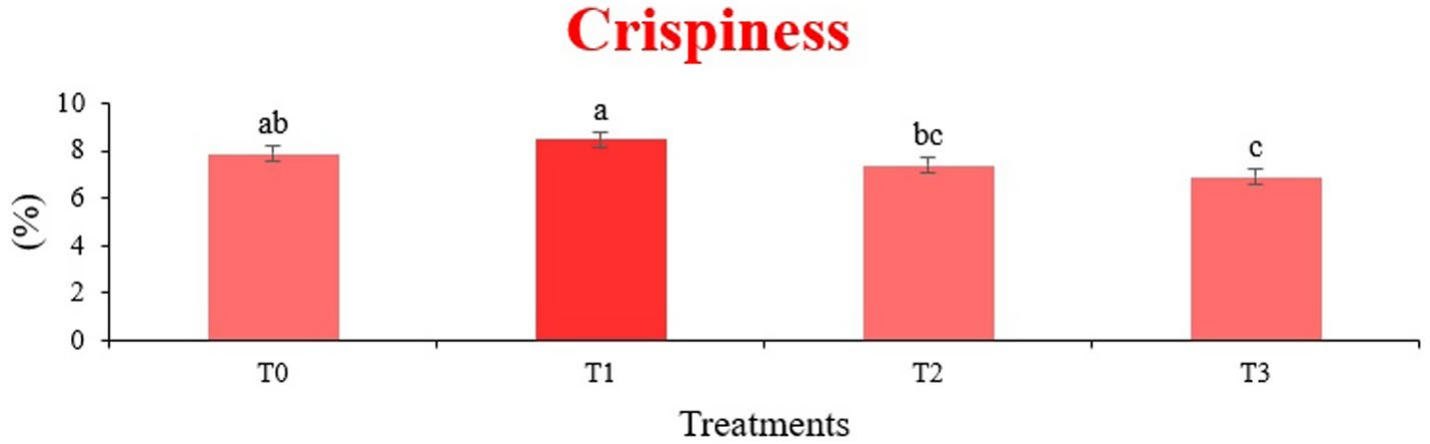
Mean results of crispiness in sensory evaluation of four different treatments of fried sorghum-soy chips.

**Figure 3:**
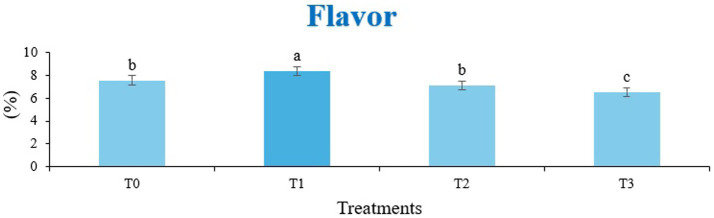
Mean results of flavor in sensory evaluation of four different treatments of fried sorghum-soy chips.

**Figure 4:**
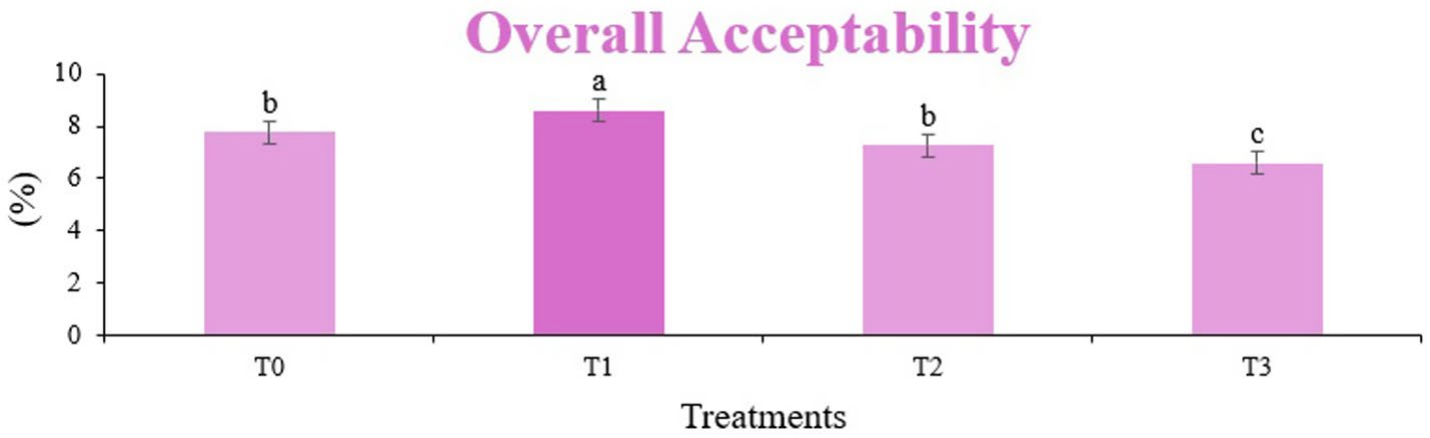
Mean results of overall acceptability in sensory evaluation of four different treatments of baked sorghum-soy chips.

**Figure 5:**
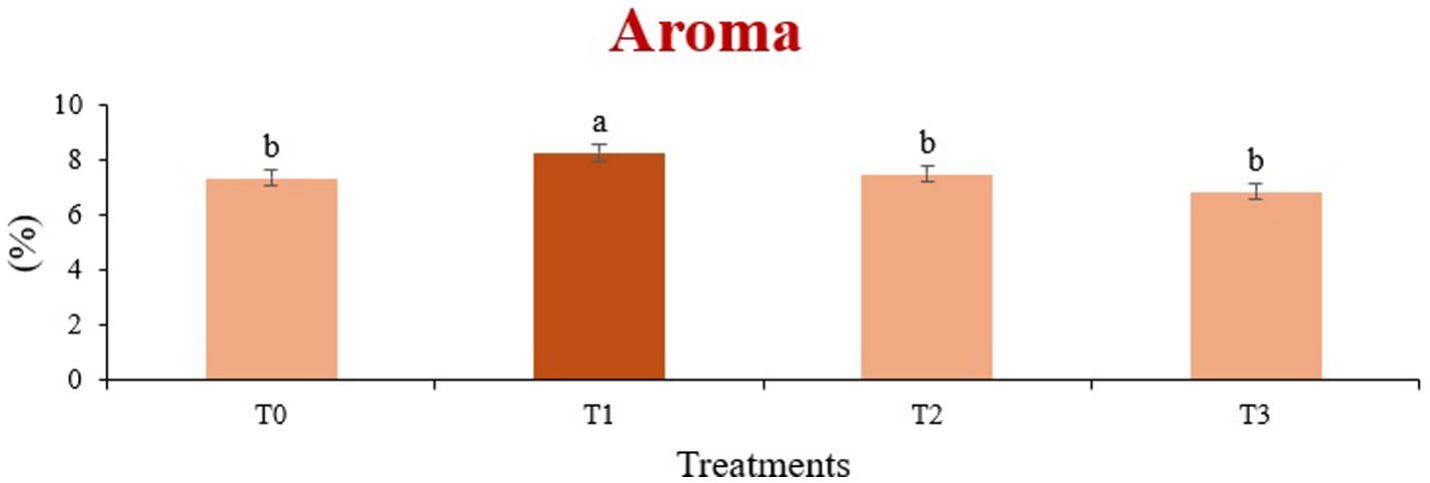
Mean results of aroma in sensory evaluation of four different treatments of fried sorghum-soy chips.

**Figure 6:**
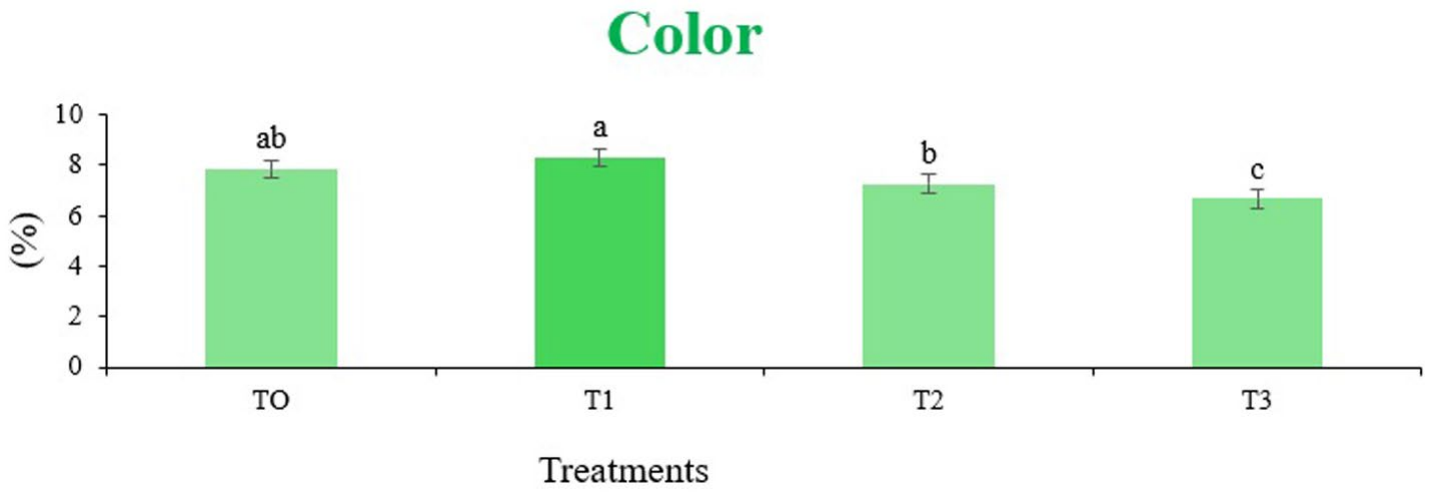
Mean results of color in sensory evaluation of four different treatments of baked sorghum-soy chips.

**Figure 7:**
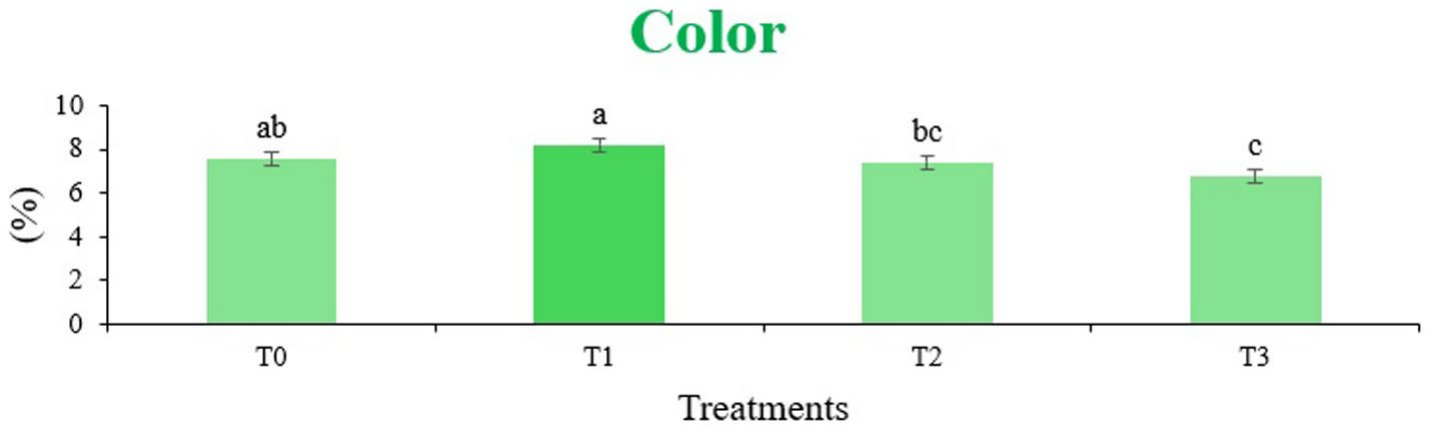
Mean results of color in sensory evaluation of four different treatments of fried sorghum-soy chips.

**Figure 8:**
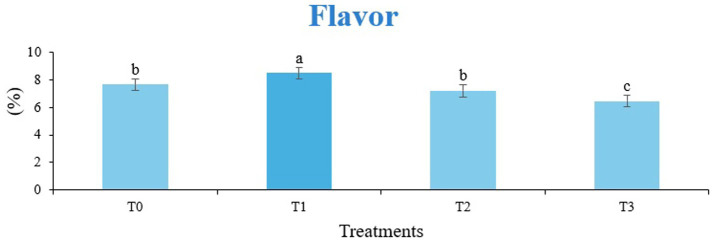
Mean results of flavor in sensory evaluation of four different treatments of baked sorghum-soy chips.

**Figure 9:**
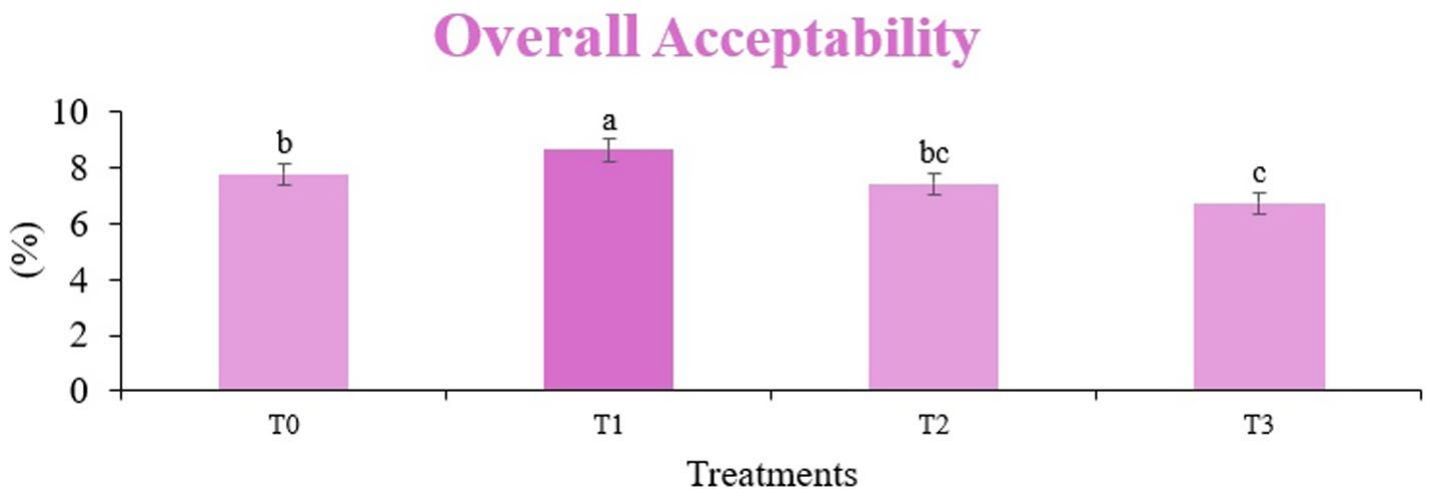
Mean results of overall acceptability in sensory evaluation of four different treatments of fried sorghum-soy chips.

**Figure 10:**
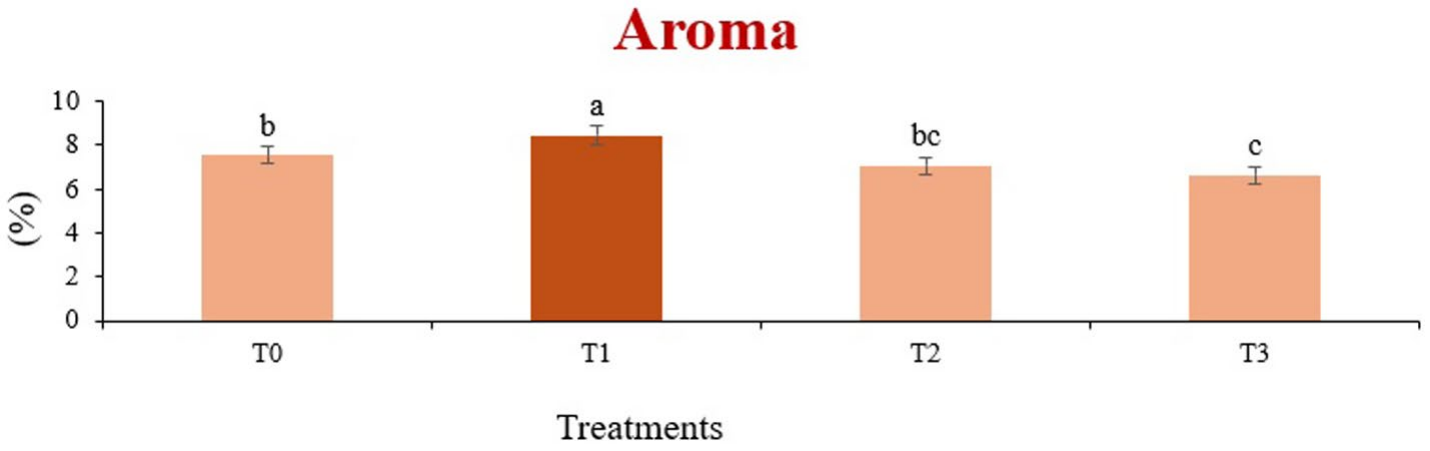
Mean results of aroma in sensory evaluation of four different treatments of baked sorghum-soy chips.

The sensory quality of protein-enriched sorghum-soy chips was evaluated to determine the influence of formulation ratios and cooking methods on consumer acceptability. As presented in Tables 14, 15, the T_1_ formulation achieved the highest scores for appearance, texture, flavor, and overall acceptability in both baked and fried products. Baked chips were preferred for their lighter crispness (Figure 1) and perceived health benefits, whereas fried chips offered superior crispiness (Figure 2) and an intensified flavor profile (Figure 3). Panel evaluations revealed that T_1_ baked chips attained an overall acceptability score (Figure 4) of 8.60 ± 8.66, which could be attributed to their balanced nutty flavor and appealing crunch. Fried chips scored slightly higher (8.63 ± 8.71), reflecting their enhanced crispiness and aroma (Figure 5) derived from Maillard browning during frying. These outcomes surpass the sensory scores reported for finger millet-fortified gluten-free chips (6.07–6.17) by Zahran et al. (78), where higher millet inclusion imparted dense textures and bitter undertones that reduced acceptability. Similarly, Liang et al. (79) observed declining sensory scores in soy protein isolate-enriched wheat noodles due to beany off-flavors and textural changes, issues that effectively mitigated in the sorghum-soy T1 blend. Superior texture ratings (8.20 ± 8.26 baked, 8.46 ± 8.55 fried) highlight T_1_’s optimized structural properties. In contrast, Ayo-Omogie (80) reported reduced flavor and texture scores in sorghum cookies with unripe banana and sesame seed meal at high substitution levels. Giri and Sakhale (81) identified textural challenges in gluten-free sweet potato spaghetti due to increased hardness. Furthermore, Cervini et al. (82) documented stickiness and softness in resistant starch-fortified gluten-free pasta, resulting in lower acceptability (5.3–6.3). These findings emphasize that the synergistic blend of soybean and sorghum in T_1_ supports desirable flavor, texture, and aroma for snack applications. Baking allows for a more uniform browning due to the Maillard reaction without the interference of oil, resulting in an attractive golden color for chewing (Figure 6). Frying can sometimes produce uneven colors (Figure 7) because of the rapid surface cooking. The flavor from baking is more natural (Figure 8) than that from sorghum and soy, while frying tends to cover up most hints with the oil flavor; deeper roasted undertones are acquired by baked chips. Frying removes moisture quickly, creating a crunchier texture (Figure 9). In contrast, baking may not achieve such a crunchy texture since it dehydrates more slowly. Baking maximizes dry heat to enhance compounds from soy and sorghum. Fried aroma is usually suppressed by oil absorption and volatilization of some key scent molecules. All in all, baked chips have been supremely excellent in flavor as well as in aroma (Figure 10), but fried chips probably carried the total weight, favoring consumer preference, owing to the factor of texture, which plays a big role in snack satisfaction.

**Table 14:** Results of sensory evaluation of baked sorghum-soy chips of different treatments.

Treatments	Color	Flavor	Crispiness	Aroma	Overall acceptability
T_0_	7.83 ± 7.91^ab^	7.66 ± 7.72^b^	7.46 ± 7.55^b^	7.53 ± 7.61^b^	7.76 ± 7.85^b^
T_1_	8.30 ± 8.36^a^	8.50 ± 8.56^a^	8.20 ± 8.26^a^	8.46 ± 8.55^a^	8.60 ± 8.66^a^
T_2_	7.26 ± 7.35^b^	7.20 ± 7.30^b^	6.93 ± 7.01^bc^	7.06 ± 7.15^bc^	7.266 ± 7.35^b^
T_3_	6.66 ± 6.72^c^	6.46 ± 6.55^c^	6.56 ± 6.65^c^	6.63 ± 6.71^c^	6.60 ± 6.70^c^

The mean values carrying the same letters in a column are not significantly different.

**Table 15:** Results of sensory evaluation of fried sorghum-soy chips of different treatments.

Treatments	Color	Flavor	Crispiness	Aroma	Overall acceptability
T_0_	7.56 ± 7.65^ab^	7.56 ± 7.62^b^	7.86 ± 7.95^ab^	7.36 ± 7.42^b^	7.76 ± 7.85^b^
T_1_	8.20 ± 8.30^a^	8.40 ± 8.50^a^	8.46 ± 8.55^a^	8.26 ± 8.35^a^	8.63 ± 8.71^a^
T_2_	7.40 ± 7.53^bc^	7.13 ± 7.17^b^	7.40 ± 7.46^bc^	7.50 ± 7.53^b^	7.43 ± 7.54^bc^
T_3_	6.76 ± 6.88^c^	6.53 ± 6.61^c^	6.90 ± 7.033^c^	6.86 ± 6.95^b^	6.73 ± 6.81^c^

The mean values carrying the same letters in a column are not significantly different.

**Figure 11:**
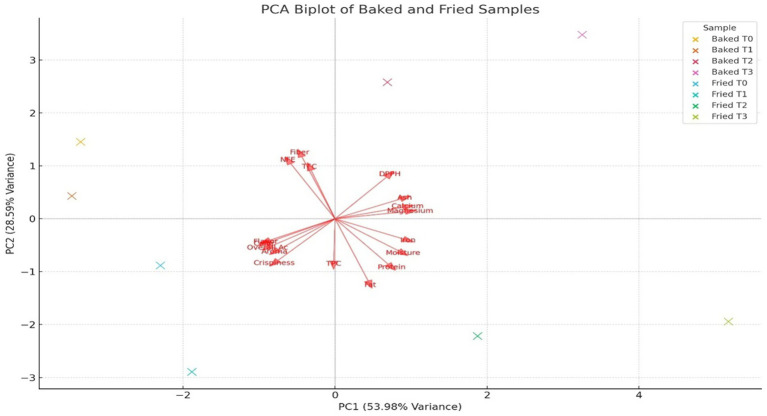
A biplot of principal component analysis of sensory attributes of both baked and fried sorghum soy chips.

The principal component analysis (PCA) biplot (Figure 11) cumulatively accounted for 82.57% of the total variance (PC1: 53.98%, PC2: 28.59%), which visually depicts the multivariate relationships on sensory and nutritional attributes of sorghum-soy chips subjected to two different processing methods: baking and frying. PC1 was the strongest axis of variation, distinguishing samples primarily through their sensory quality and proximate composition. Positive correlations were found for fried treatments, more specifically for Fried T1, along with attributes crispness, flavor, and overall acceptability. Hence, frying imparts greater sensory appeal via improved texture and palatability. On the other hand, baked treatments, specifically Baked T1 and Baked T2, were more associated with nutritional parameters such as dietary fiber, ash content, and calcium, magnesium, as well as antioxidant capacity (DPPH), probably suggesting that baking well preserves or augments the nutritional integrity of the product.

Further contribution of sample differentiation can be attributed to PC2 with respect to mineral variance and physical appearance. From the spatial distribution of vectors in the biplot, it can be deduced that baked samples are more related to components that promote health, while fried samples have an even more favorable compositional reception in terms of consumers. Therefore, there is a compromise between nutritional quality and sensory acceptability of baked over fried sorghum-soy chips, with the processing method having a significant impact on the overall profile of the product.

## 4 Conclusion

This study demonstrated the superior nutritional quality, functional potential, and sensory acceptability of sorghum-soy chips, supporting their suitability as low-glycemic, protein-rich snack alternatives. Incorporating soybean flour in varying ratios significantly enhanced the chips’ protein content, amino acid balance, and mineral density, indicating their potential in addressing protein–energy malnutrition. Sensory evaluation revealed that treatments with higher soybean content (T_1_) achieved the most favorable scores for color, flavor, aroma, crispiness, and overall acceptability. Proximate analysis confirmed a balanced nutritional profile, while mineral analysis highlighted calcium, iron, and magnesium levels beneficial for cognitive and physical development in school-aged children. Both baked and fried methods produced chips with desirable texture and shelf stability; however, baked chips exhibited slightly lower fat content, aligning with consumer preferences for healthier snack options. The low glycemic index of these chips further emphasizes their appropriateness for health-conscious individuals and those managing blood glucose levels. Utilizing locally sourced sorghum and soybeans not only contributes to sustainable food systems but also provides an affordable, nutrient-dense snack option for vulnerable populations. These findings align with current trends in functional food innovation, offering a promising strategy to diversify diets and combat malnutrition. Future research should explore shelf-life stability and market feasibility to support large-scale production of such sustainable snacks. These sorghum-soy composite chips have great potential for commercial options, especially in markets highly interested in cost-effective yet nutrient-rich alternatives for snacks. In addition, for both soy and sorghum, these crops are plentiful suppliers where the production can be scaled with minimal constraints on the supply chain. Nutritionally, chips have a good glycemic profile and a progressive increase in antioxidant potential with rising soy levels. These characteristics show promise for moderating postprandial glycemic response and oxidative stress conditions concerning metabolic health and chronic disease prevention. While both baking and frying are alternative processing treatments, they contain a wealth of possibilities for innovating product quality and diversity in most sustainable practices.

## Data Availability

The raw data supporting the conclusions of this article will be made available by the authors, without undue reservation.
